# Roles of Perivascular Adipose Tissue in the Pathogenesis of Atherosclerosis

**DOI:** 10.3389/fphys.2018.00003

**Published:** 2018-02-13

**Authors:** Kimie Tanaka, Masataka Sata

**Affiliations:** ^1^Division for Health Service Promotion, The University of Tokyo, Tokyo, Japan; ^2^Department of Cardiovascular Medicine, Institute of Biomedical Sciences, Tokushima University Graduate School, Tokushima, Japan

**Keywords:** perivascular adipose tissue, atherosclerosis, epicardial adipose tissue, inflammation, adipocytokine

## Abstract

Traditionally, it is believed that white adipose tissues serve as energy storage, heat insulation, and mechanical cushion, whereas non-shivering thermogenesis occurs in brown adipose tissue. Recent evidence revealed that adipose tissue secretes many types of cytokines, called as adipocytokines, which modulate glucose metabolism, lipid profile, appetite, fibrinolysis, blood pressure, and inflammation. Most of the arteries are surrounded by perivascular adipose tissue (PVAT). PVAT has been thought to be simply a structurally supportive tissue for vasculature. However, recent studies showed that PVAT influences vasodilation and vasocontraction, suggesting that PVAT regulates vascular tone and diameter. Adipocytokines secreted from PVAT appear to have direct access to the adjacent arterial wall by diffusion or via vasa vasorum. In fact, PVAT around atherosclerotic lesions and mechanically-injured arteries displayed inflammatory cytokine profiles, suggesting that PVAT functions to promote vascular lesion formation. Many clinical studies revealed that increased accumulation of epicardial adipose tissue (EAT), which surrounds coronary arteries, is associated with coronary artery disease. In this review article, we will summarize recent findings about potential roles of PVAT in the pathogenesis of atherosclerosis, particularly focusing on a series of basic and clinical studies from our laboratory.

## Perivascular adipose tissue and adipocytokines

It has been believed that atherosclerotic process is initiated by endothelial injury, followed by inflammatory cells infiltration into the subendothelial layer. Thus, inflammation spreads from the inside toward the outside of the artery (Ross, 1999). On the other hand, it is assumed that inflammatory process also progresses from the outside toward the inside. Most of the arteries surrounded by perivascular adipose tissue (PVAT). Aorta has abundant PVAT (Szasz et al., 2013), while PVAT is not detected around cerebral arteries and microvessels. There is no distinct borderline between arterial adventitia and PVAT. PVAT has been considered as simply a supporting organ of vasculature.

**Figure 1:**
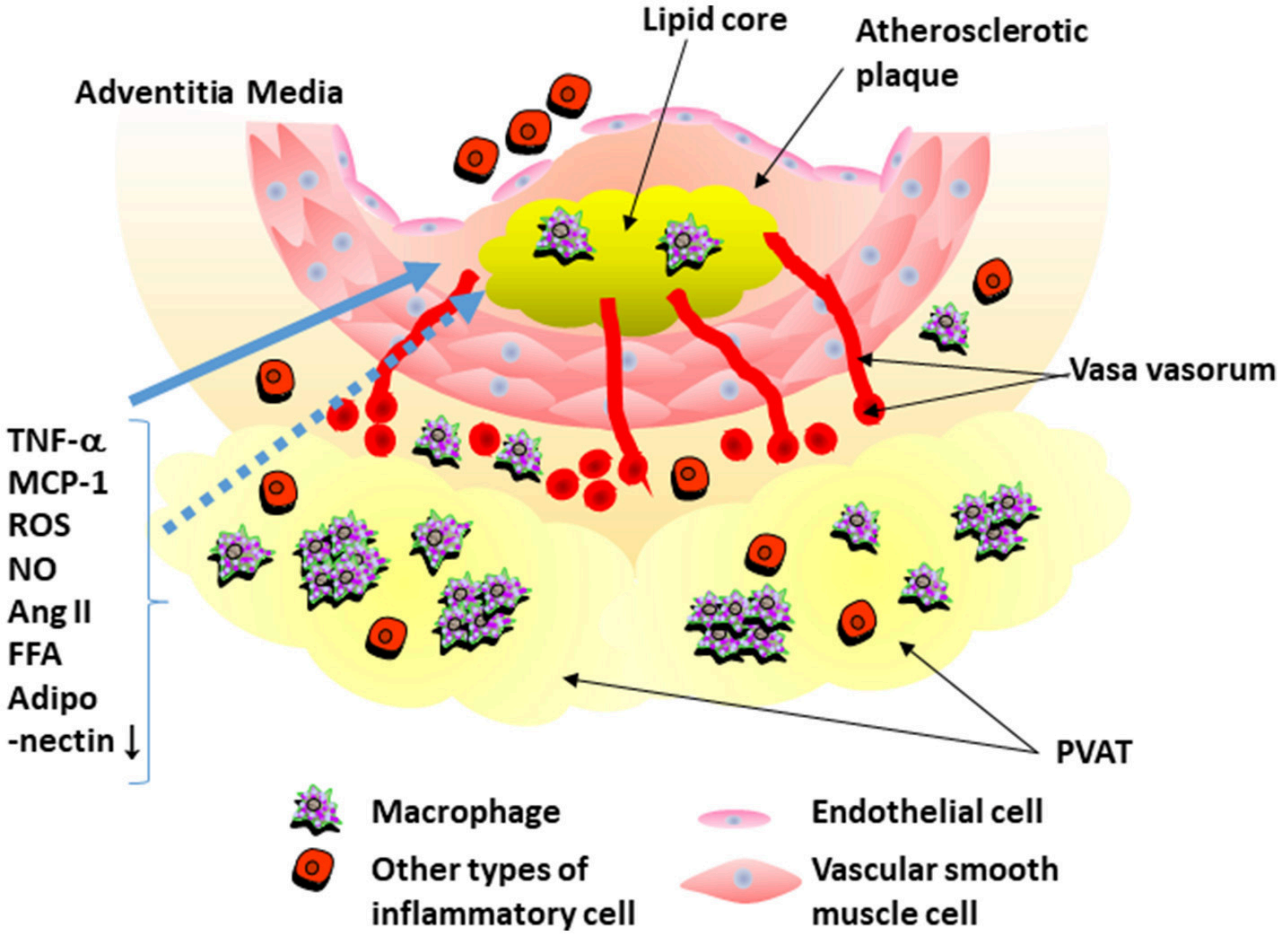
Interaction of perivascular adipose tissue and atherosclerotic plaque. Perivascular adipose tissue (PVAT) secretes inflammatory cytokines that recruit inflammatory cells into PVAT. PVAT also secrets reactive oxygen spices (ROS), nitric oxide (NO), angiotensin II, and free fatty acid (FFA). Expression of adiponectin is decreased in PVAT around atherosclerotic plaque. Adipocytokines secreted from PVAT appear to have direct access to the adjacent arterial wall by diffusion or via vasa vasorum. TNF-α, tumor necrosis factor-α; Ang II, angiotensin II; MCP-1, monocyte chemoattractant protein-1.

In 1991, Soltis and Cassis demonstrated that perivascular adipose tissue significantly influences vascular responsiveness to contractile stimuli using rat aortic rings with or without surrounding adipose tissues (Soltis and Cassis, 1991). This study suggested that PVAT potentially serves as a regulator of vascular responsiveness (Soltis and Cassis, 1991). Recently, adipose tissue have received a lot of attention as endocrine organ. It was reported that adipocytes secrete numerous kinds of inflammatory and anti-inflammatory cytokines, called as “adipocytokines,” such as tumor necrosis factor (TNF)-α and adiponectin (Matsuzawa et al., 2004). Analyses of human samples showed that PVAT around the coronary arteries, like other adipose tissues, expresses inflammatory cytokines, and chemokines (Mazurek et al., 2003; Henrichot et al., 2005). It was also reported that PVAT secretes reactive oxygen spices, nitric oxide, angiotensin II, and free fatty acid (Szasz et al., 2013). Adipocytokines secreted from PVAT appear to have direct access to the adjacent arterial wall via diffusion or vasa vasorum (Sacks and Fain, 2007; Tanaka et al., 2011) (Figure 1).

## Influence of PVAT on endothelial function and vascular lesion formation: lessons from animal models

**Figure 2:**
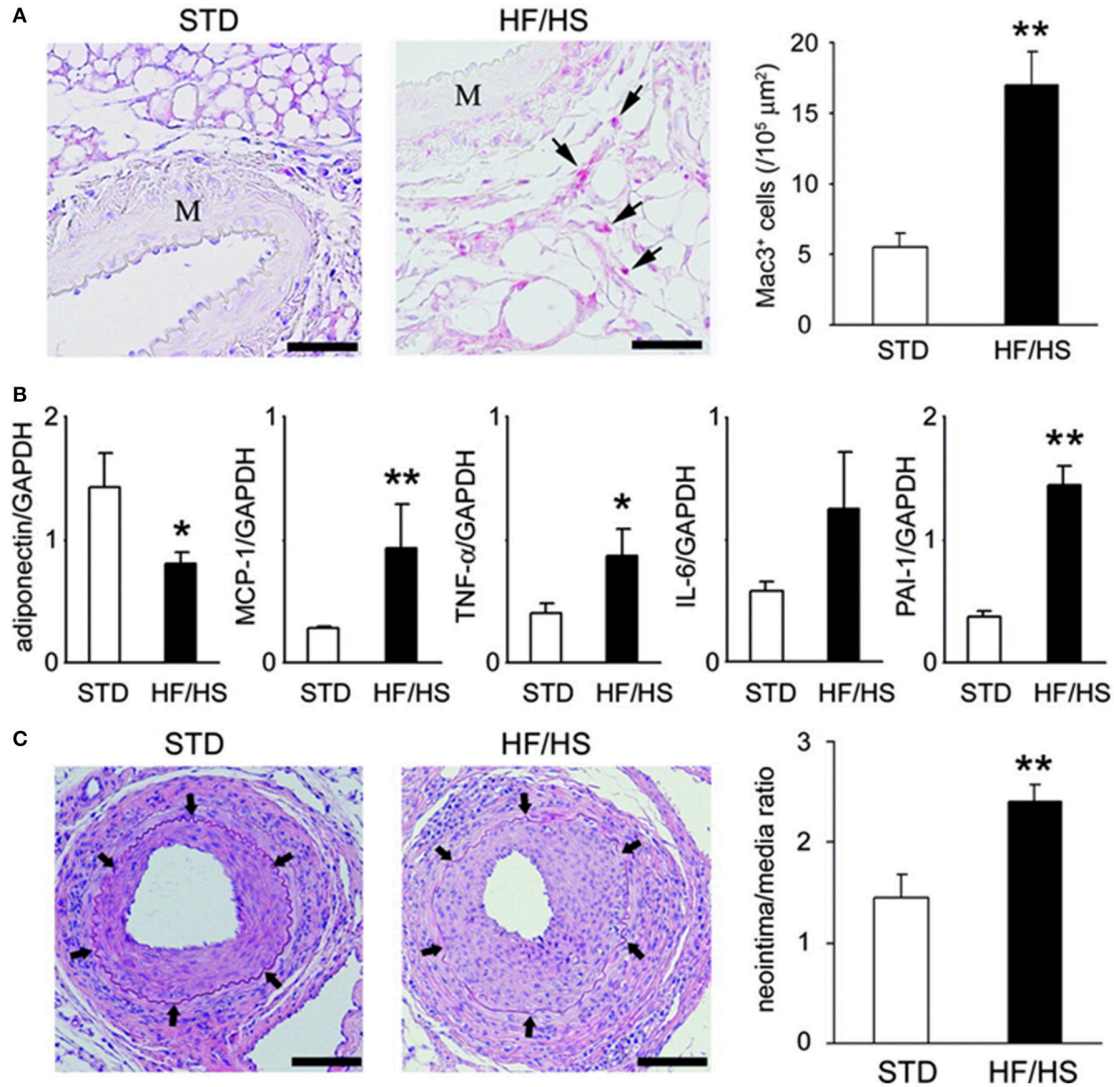
Obesity-induced inflammatory changes in periadventitial fat enhanced neointimal hyperplasia. **(A)** Obesity-induced accumulation of inflammatory cells in periadventitial fat. Immunohistochemical analysis showed accumulation of Mac3-positive macrophages (arrows) within periadventitial fat in obese mice. Scale bar: 50 μm. Results are expressed as mean ± SEM. ^**^P < 0.01. M indicates media of femoral artery. **(B)** Expression of mRNA in periadventitial fat around femoral artery from STD (standard diet) (*n* = 6) and HF/HS (high fat/high sucrose diet) WT C57BL6 mice. Expression level was assessed by real-time PCR normalized to each GAPDH level. Results are expressed as means ± SEM. ^*^P < 0.05, ^**^P < 0.01. **(C)** Hematoxylin/eosin-stained sections of femoral arteries from mice fed on STD or HF/HS diet 4 weeks after endovascular injury. Arrows indicate internal elastic lamina. Scale bar: 100 μm. Morphometric analysis of injured femoral arteries in lean (*n* = 7) and obese (*n* = 6) mice 4 weeks after wire-induced injury. Results are expressed as means ± SEM. ^**^P < 0.01. All figures are cited from the reference (Takaoka et al., 2009) with permission.

We evaluated whether inflammation in PVAT affects the lesion formation after a mechanical vascular injury in murine femoral artery (Takaoka et al., 2009) (Figure 2). Wild-type (C57BL/6) mice received either a standard chow diet or a high-fat high-sucrose (HF/HS) diet. The body weight of the wild-type mice increased by 54% with a HF/HS feeding. The number of macrophages accumulated in PVAT increased by HF/HS diet. Expression of adiponectin was down-regulated, while expression of inflammatory cytokines was up-regulated in PVAT of mice fed on HF/HS diet. A wire was inserted into the femoral artery of mice to induce endothelial denudation and over-dilatation of the femoral artery. The changes in cytokine expression in PVAT around the injured artery was associated with exaggerated neointima formation at 4 weeks after the injury, suggesting that PVAT influences pathological vascular remodeling in response to mechanical vascular injury.

We also investigated an atheroprotective role of healthy PVAT by removing PVAT in mice fed on a standard diet. Removal of healthy PVAT markedly enhanced neointima formation, which was attenuated by transplantation of subcutaneous fat tissues from the mice fed on a standard diet. The results suggest an atheroprotective role of healthy PVAT. On the other hand, transplantation of subcutaneous fat from the obese mice or visceral fat failed to show atheroprotective effect. To investigate the local effects of PVAT adiponectin on vascular remodeling, recombinant adiponectin was delivered locally to the adventitial space in adiponectin-deficient mice, using gelatin hydrogel. Four weeks after endovascular injury, neointima formation was reduced by perivascular delivery of adiponectin. Taken together, it was suggested that PVAT functions to prevent lesion formation by secreting atheroprotective adipokines, such as adiponectin. However, obesity alters adipocytokine expression profiles of PVAT, resulting in enhanced neointima formation after vascular injury (Takaoka et al., 2009).

We also reported that mechanical endovascular injury alters adipocytokine expression in PVAT (Takaoka et al., 2010). A wire was inserted into the femoral artery of mice to induce endothelial denudation and over-dilatation. We found that this mechanical injury up-regulated inflammatory cytokines and down-regulated adiponectin in PVAT. These changes were attenuated in TNF-α knockout mice, suggesting that TNF-α is important to transmit endovascular injury to adipocytokine changes in PVAT (Takaoka et al., 2010).

Consistent with our studies, others reported that PVAT plays a role in the pathogenesis of vascular lesion formation. Ketonen et al. reported that obesity-induced endothelial dysfunction is caused by increased oxidative stress and enhanced expression of inflammatory cytokine in PVAT (Ketonen et al., 2010). Manka et al. reported that transplantation of PVAT from obese mice to low-density lipoprotein receptor knockout mice enhanced lesion formation with increased inflammatory cell infiltration and pathological angiogenesis in adventitia. Theses pathological effects of PVAT transplantation was attenuated when PVAT from monocyte chemoattractant protein-1 (MCP-1)-deficient mice was transplanted. These results suggest that PVAT promotes vascular lesion formation through MCP-1-dependent mechanisms (Manka et al., 2014). These animal studies indicate that obesity increases expression of inflammatory adipocytokines in PVAT, leading to endothelial dysfunction and enhanced vascular lesion formation.

## Possible roles of epicardial adipose tissue in the pathogenesis of human coronary artery disease

Epicardial adipose tissue (EAT) is assumed to secrete abundant cytokines to the adjacent coronary artery (Sacks and Fain, 2007). For example, in the patients undergoing coronary artery bypass graft (CABG) surgery, it was reported that EAT abundantly expressed interleukin (IL)-1β, IL-6, TNF-α, and MCP-1 compared to their subcutaneous adipose tissue (Mazurek et al., 2003). Baker et al. reported that the expression of adiponectin mRNA was significantly lower in EAT than in gluteal and abdominal adipose tissues (Baker et al., 2006).

**Figure 3:**
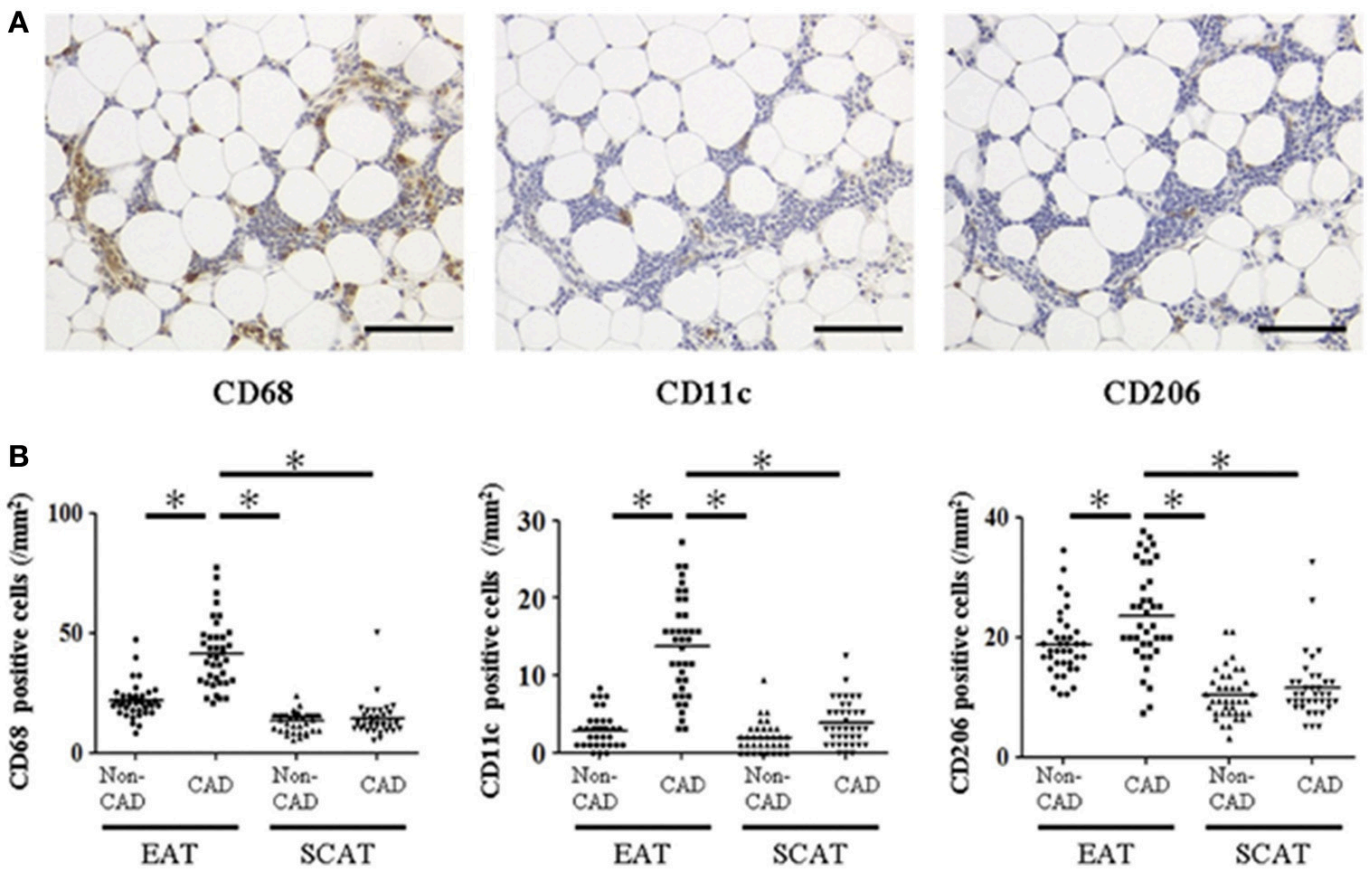
Macrophage infiltration in human epicardial adipose tissue and subcutaneous adipose tissue. **(A)** Representative images of immunohistochemical staining showing accumulation of CD68-, CD11c-, and CD206-positive cells in epicardial adipose tissue (EAT) of coronary artery disease (CAD) patient. Scale bar = 100 μm. **(B)** Cell count of accumulating macrophages. Each point represents the cell count of infiltrating macrophages (/mm^2^). Bar indicates mean. ^*^p < 0.05. SCAT, subcutaneous adipose tissue. All figures are cited from the reference (Hirata et al., 2011b) with permission.

However, it remains to be elucidated whether the potential role of chronic inflammation in EAT plays a role in the pathogenesis of coronary artery disease (CAD). Therefore, we analyzed EAT obtained during cardiac surgery (Hirata et al., 2011a,b). EAT and subcutaneous adipose tissue (SCAT) were obtained from 38 CAD patients undergoing CABG and 40 non-CAD patients undergoing valvular surgery (Hirata et al., 2011b). Expressions of IL-6 and TNF-α were significantly increased in EAT of the CAD group compared to that of the non-CAD group. There was no significant difference between the CAD and the non-CAD groups in the expression of adipocytokines in SCAT. To investigate the mechanisms by which expression of inflammatory cytokines is elevated in EAT of the CAD patients, we performed immunohistochemistry against CD68, a marker for all types of macrophages, CD11c, a marker for inflammatory M1 macrophage (Lumeng et al., 2007), and CD206, a marker for anti-inflammatory M2 macrophage (Bourlier et al., 2008) (Figure 3). CD68 positive macrophages were significantly increased in EAT of the CAD group. The ratio of CD11c/CD68-positive cells was significantly increased, and the ratio of CD206/CD68-positive cells was significantly decreased in EAT in the CAD group. These data demonstrate relative increase of M1 macrophages and relative decrease of M2 macrophages in EAT in the CAD group. The ratio of M1/M2 macrophages showed positive correlation with the severity of CAD as determined by Gensini score (Gensini, 1983). These results suggested that the chronic inflammation and macrophage polarization in EAT would play a pathological role in human coronary atherosclerosis.

Recently, it was reported that the expression of omentin was detected in EAT. Omentin, also known as interectin-1, is one of the recently identified adipocytokines (Harada et al., 2016). Omentin is expressed abundantly in omentum adipose tissue and is considered to have cardiovascular protective effects like adiponectin. It is known that omentin expression decreases in the milieus of diabetes mellitus or obesity (Shibata et al., 2017). Harada et al. analyzed EAT and SCAT from 15 non-obese CAD patients and 10 non-obese and non-CAD patients. Omentin expression increased in the EAT of non-obese CAD patients, despite a decrease in plasma levels. These results indicated that omentin expression in EAT may play a certain role in the pathogenesis of CAD (Harada et al., 2016).

## Epicardial adipose tissue volume and coronary artery disease

More than 200 years ago, an autopsy report of the case of a patient, who died in 1801, already described that CAD was combined with unusual accumulation of fat about the heart (Warren, 1962). Recently, many groups including us suggested that CAD is associated with increased EAT volume (Dagvasumberel et al., 2012). EAT volume can be quantified by coronary CT, echocardiography and MRI (magnetic resonance imaging). Konishi et al. measured “pericardial adipose tissue” volume by 64-slice CT, and suggested that CAD is more highly associated with pericardial fat volume than abdominal obesity (Konishi et al., 2010). Other studies also suggested that EAT volume may predict the severity of coronary atherosclerotic lesions and the clinical prognoses (Dagvasumberel et al., 2012). Moreover, a recent study indicated that EAT volume predicts fatal and non-fatal cardiac events independently of the classical coronary risk factors (Mahabadi et al., 2013). We also investigated the impact of EAT volume on CAD (Dagvasumberel et al., 2012). Multivariate analysis indicated that EAT volume index [EAT volume/body surface area (BSA)] were significant CAD predictors in men, whereas BMI, age, presence of hypertension, diabetes mellitus, and hyperlipidemia were not associated with the presence of CAD.

Previous studies suggested that increased visceral adipose tissue (VAT) is associated with CAD. For example, the prospective long-term follow-up of the Framingham Heart Study showed that VAT was an independent predictor of incident of cardiovascular disease (Britton et al., 2013). Accumulating evidence suggests that inflammatory cells can be observed in VAT than in SCAT and that VAT secretes more inflammatory adipocytokines than SCAT (Ibrahim, 2010). It is also suggested that VAT adipocytes plays more important roles in the pathogenesis of insulin resistance than SCAT adipocytes does (Ibrahim, 2010). Thus, it is likely that VAT influences vascular function and atherosclerosis than SCAT does.

**Figure 4:**
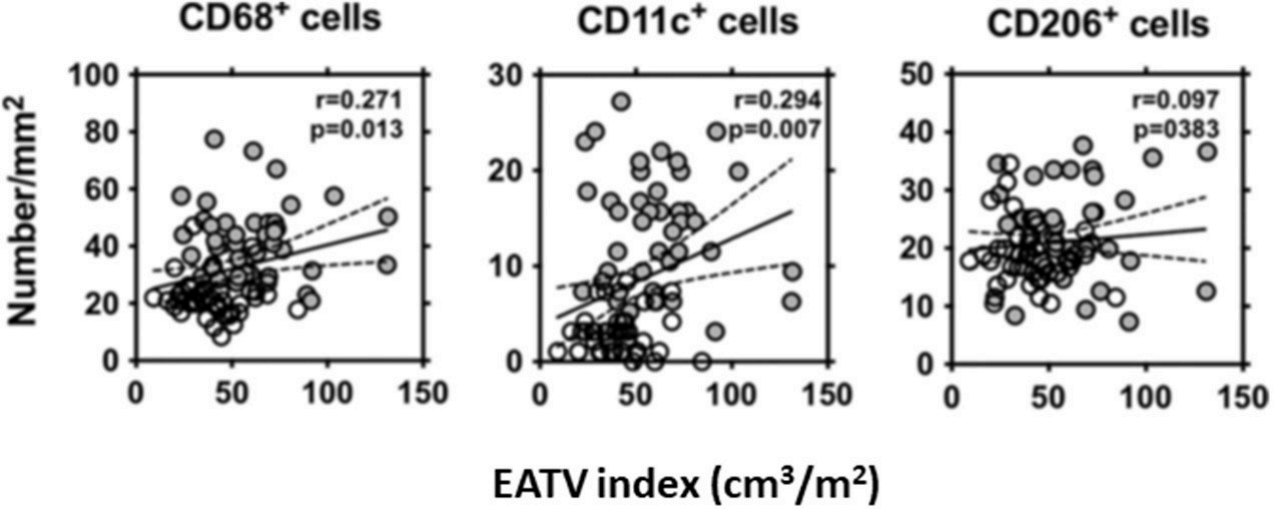
Correlation between the EAT volume index and the number of CD68+, CD11c+, and CD206+ cells in EAT. The EAT volume index was positively correlated with the numbers of CD68+, and CD11c+ cells in EAT in the patients who underwent non-coronary (◦) or coronary (•) surgery. Liner regression analysis was made in a combined group, including non-coronary artery disease (CAD) and CAD subjects. *R* and *P*-values are shown. EATV index, epicardial adipose tissue volume index. All figures cited from the reference (Shimabukuro et al., 2013) with permission.

We collected EAT and SCAT from 50 CAD patients and 50 non-CAD patients who underwent elective cardiac surgery. We evaluated the polarity of the accumulated macrophages in adipose tissue by immunohistochemical staining with the antibodies for CD68, CD11c, and CD206 and compared them with EAT volume index (Figure 4) (Shimabukuro et al., 2013). We found that EAT volume index was a significant prognostic factor to predict CAD. There were positive correlations between EAT volume index and the numbers of CD68 and CD11c positive M1 macrophage, and the expressions of inflammatory cytokines such as IL-1β, and negatively correlated with adiponectin expression in EAT (Shimabukuro et al., 2013). A multivariate analysis model revealed that number of CD68 (+) cells and IL-1β, and adiponectin expression in EAT strongly predicted CAD. These results indicated that EAT volume, macrophage infiltration, and adipocytokine signals in EAT are closely associated with CAD and that EAT volume and adipocytokine imbalance play critical roles in the pathogenesis of human coronary atherosclerosis.

Recent reports suggested that EAT accumulation is associated with not only coronary artery disease but also atrial fibrillation (AF) (Soeki and Sata, 2012). In Framingham heart cohort, EAT volume was measured by CT in 3217 subjects, and it was suggested that EAT volume was an independent risk factor of AF after adjusting other risk factors such as hypertension, PR interval, and body mass index (BMI) (Thanassoulis et al., 2010). Another study demonstrated that peri-atrial EAT volume was associated with new-onset AF in patients with CAD, independent of enlargement of the left atrium (Nakanishi et al., 2012). It is assumed that inflammatory cytokines secreted from peri-atrial EAT promote fibrotic remodeling of atrial myocardium, leading to AF (Hatem et al., 2016).

## How to evaluate epicardial adiposity?

**Figure 5:**
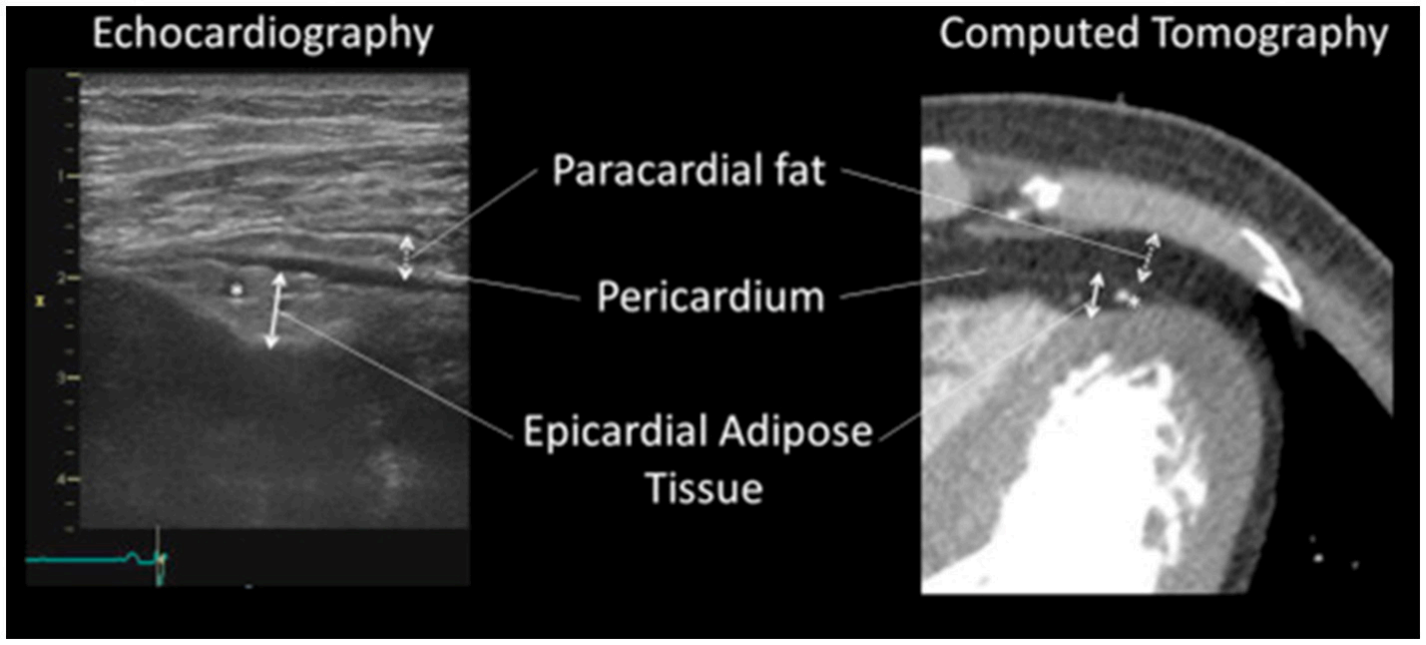
Identification of epicardial adipose tissue and paracardial adipose tissue. Identification of epicardial adipose tissue and paracardial adipose tissue by echocardiography **(Left)** and contrast enhanced 320-slice multi-detector computed tomography **(Right)**. The asterisks (^*^) indicate left descending coronary artery. “Pericardial adipose tissue” includes both epicardial adipose tissue (located within or deep into the pericardium) and paracardial adipose tissue (located superficial to the pericardium). Sometimes, pericardial adipose tissue is referred to as paracardial adipose tissue. All figures cited from the reference (Yamada and Sata, 2015) without modification.

Although many groups have investigated correlation between the EAT volume and coronary atherosclerotic lesions, there might be a confusion in definition of fat depots around the heart (Yamada and Sata, 2015). EAT, or subepicardial adipose tissue, located inside the parietal pericardium, have a direct contact with coronary artery (Figure 5). On the other hand, adipose tissue located outside the parietal pericardium is called as paracardial adipose tissue (PAT). PAT is also called as thoracic or intrathoracic adipose tissue. There is a difference in the definition of “pericardial adipose tissue.” In some studies, PAT was described as “pericardial adipose tissue,” whereas EAT or EAT together with PAT were described as “pericardial adipose tissue” in other studies (Yamada and Sata, 2015). EAT shares coronary circulation with cardiac myocardium, while PAT is perfused by non-coronary source. Thus, EAT and PAT are distinct fats with different impacts on coronary atherosclerosis (Yamada and Sata, 2015). We would like to define that “pericardial adipose tissue” means “epicardial adipose tissue” plus “paracardial adipose tissue.”

To evaluate EAT accumulation, different groups use EAT volume (Konishi et al., 2010; Mahabadi et al., 2013) or EAT volume index, which is EAT volume divided by BSA. We found that the EAT volume was higher in men than in women, but the mean EAT volume/height and EAT volume/BSA were comparable (Dagvasumberel et al., 2012). Therefore, it is likely that EAT volume index might be a preferable parameter (Shimabukuro et al., 2013).

El Khoudary et al. assessed whether volumes of heart fat depots (EAT and PAT) were associated with coronary artery calcification (CAC) in women at midlife and whether these associations were modified by menopausal status and estradiol levels (El Khoudary et al., 2017). Volumes of PAT and EAT increased after menopause. Of note, estradiol decline was associated with PAT volume, but not EAT volume, suggesting that menopause has something to do with PAT accumulation. CAC measures were associate with EAT volume. Menopausal status or estradiol did not modify this association. In contrast, menopausal status significantly modified association between PAT and CAC measures. It was reported that PAT might be a risk factor for coronary artery disease in menopausal women. It was suggested that PAT depot need to be monitored and would be a target for intervention in women at midlife (El Khoudary et al., 2017).

Many groups measured EAT thickness using echo-cardiography and reported that EAT thickness was greater in CAD patients than in non-CAD patients (Iacobellis, 2015). Recently, we developed a new method to evaluate EAT thickness (Hirata et al., 2015). We evaluated EAT thickness at anterior interventricular groove (EAT-AIG) and at anterior right ventricle (EAT-RV) of 311 patients by echocardiography using a high frequency linear probe. EAT-AIG had a strong correlation with EAT volume evaluated by coronary CT. Both EAT-AIG and EAT-RV of CAD patients were greater than those of non-CAD patients. EAT-AIG was more strongly correlated with CAD as determined by the receiver operating characteristics curve analysis. It was suggested that we may be able to predict CAD with high sensitivity and specificity by evaluating EAT thickness by the non-invasive echocardiography using the high frequent linear probe (Hirata et al., 2015).

Nerlekar at al. performed meta-analysis to assess the association between EAT and high-risk plaque (HRP) (Nerlekar et al., 2017). Nine studies (*n* = 3,772 patients) were included with seven measuring EAT volume by CT and two measuring EAT thickness by echocardiography. Increase in EAT volume or thickness was associated with the presence of HRP. Increasing EAT volume has a significant association with HRP. However, EAT thickness had no significant association with HRP. This analysis included only two studies evaluating EAT thickness by echocardiography. Further investigation is required to establish clinical significance of evaluating EAT thickness to predict the existence of HRP.

Besides coronary CT and echocardiography, it was reported that EAT volume can be evaluated by MRI (Levelt et al., 2016). There is no standard method to evaluate EAT volume to predict coronary atherosclerosis disease. It is hoped that EAT can be evaluated precisely and easily using appropriate modalities.

## Can PVAT be reduced to prevent cardiovascular events?

It is very important to clarify whether modification of life-style or medication can reduce PVAT, leading to phenotypic improvement of inflammatory PVAT. It was reported that EAT was decreased by aerobic exercise for 12 weeks in obese middle-aged men (Kim et al., 1985). Interestingly, reduction in EAT volume showed linear correlation with reduction in VAT volume (Kim et al., 1985).

In a sub-analysis of BELLES (Beyond Endorsed Lipid Lowering with Electron Beam Tomography Scanning) trial, in which the effect of the moderate and the aggressive doses of statin therapy to coronary calcification were tested in 615 hyperlipidemic post-menopausal women, EAT volume was measured by CT in the intensive therapy group (atorvastatin 80 mg) and the moderate therapy group (pravastatin 40 mg). One year later, reduction of EAT volume was observed in the intensive and the moderate therapy groups. Decrease in EAT volume was statistically significant in the intensive therapy group, but not in the moderate therapy group. Interestingly, EAT volume reduction showed no correlation with the degree of lipid lowering. These results suggested that, in post-menopausal women, statin therapy decreased EAT volume especially in the intensive therapy group and that the effect of statin was not associated with the LDL lowering effect. Anti-inflammatory pleiotropic effects of statin might be related to this effect (Alexopoulos et al., 2013).

Recently, it was reported that sodium-glucose co-transporter 2 (SGLT2) inhibitors (ipragliflozin, luseogliflozin, and canagliflozin) reduced the EAT (Bouchi et al., 2017; Fukuda et al., 2017; Yagi et al., 2017) as well as the abdominal visceral fat (Tosaki et al., 2017) in type 2 diabetes patients. Recent randomized clinical trials showed that use of SGLT2 inhibitors (canagliflozin and empagliflozin) decreases mortality and morbidity of cardiovascular diseases in diabetic patients (Zinman et al., 2015; Neal et al., 2017). If SGLT2 inhibitors can decrease EAT volume as well as other adipose tissues, it is assumed that reduction in EAT could lead to cardiovascular protective effects by SGLT2 inhibitors, which have been proved by recent randomized clinical trials (Heerspink et al., 2016; Rajasekeran et al., 2016).

It remains to be clarified whether the decrease of EAT by any therapeutic interventions can inhibit progression of coronary lesions or the occurrence of coronary events. Further studies will clarify whether reduction in EAT volume could be a therapeutic target to prevent cardiovascular events.

## Conclusions

PVAT has been considered to secrete humoral factors, influencing the function and the lesion formation of the adjacent artery. EAT volume can be measured by CT, echocardiography and MRI, which are commonly used in clinical practices. Numerous imaging studies suggested that increased EAT volume is associated with CAD. Almost all the data presented is correlative. It remains to be elucidated whether the reduction of EAT volume would be effective in prevention of cardiovascular events. Future studies will clarify more in detail the roles of PVAT in the pathogenesis of CAD. EAT would be a useful biomarker in the diagnosis of CAD and would be a good therapeutic target.

## Disclosures

MS received research funding from Takeda, Tanabe-Mitsubishi, Astellas, Daiichi-Sankyo, MSD, Bayer Healthcare, and Ono, and lecture fees from Takeda, Boehringer Ingelheim, Bayer Healthcare, Mochida, Astellas, Tanabe-Mitsubishi, Novartis, AstraZeneca, MSD, and Shionogi. The Department of Cardio-Diabetes Medicine, Tokushima University Graduate School, is supported in part by unrestricted research grants from Boehringer Ingelheim, Tanabe-Mitsubishi, Kowa, and Actelion.

## Author contributions

All authors listed have made a substantial, direct and intellectual contribution to the work, and approved it for publication.

### Conflict of interest statement

The authors declare that the research was conducted in the absence of any commercial or financial relationships that could be construed as a potential conflict of interest.
